# Critical evaluation of methods used to determine amplification efficiency refutes the exponential character of real-time PCR

**DOI:** 10.1186/1471-2199-9-96

**Published:** 2008-10-30

**Authors:** Robert G Rutledge, Don Stewart

**Affiliations:** 1 Natural Resources Canada, Canadian Forest Service, 1055 du PEPS, Quebec, Quebec G1V 4C7, Canada

## Abstract

**Background:**

The challenge of determining amplification efficiency has long been a predominant aspect of implementing real-time qPCR, playing a critical role in the accuracy and reliability that can be achieved. Based upon analysis of amplification profile position, standard curves are currently the gold standard for amplification efficiency determination. However, in addition to being highly resource intensive, the efficacy of this approach is limited by the necessary assumption that all samples are amplified with the same efficiency as predicted by a standard curve. These limitations have driven efforts to develop methods for determining amplification efficiency by analyzing the fluorescence readings from individual amplification reactions. The most prominent approach is based on analysis of the "log-linear region", founded upon the presumption that amplification efficiency is constant within this region. Nevertheless, a recently developed sigmoidal model has provided new insights that challenge such historically held views, dictating that amplification efficiency is not only dynamic, but is linearly coupled to amplicon DNA quantity. Called "linear regression of efficiency" or LRE, this kinetic-based approach redefines amplification efficiency as the maximal efficiency (E_max_) generated at the onset of thermocycling.

**Results:**

This study presents a critical evaluation of amplification efficiency determination, which reveals that potentially large underestimations occur when exponential mathematics is applied to the log-linear region. This discrepancy was found to stem from misinterpreting the origin of the log-linear region, which is derived not from an invariant amplification efficiency, but rather from an exponential loss in amplification rate. In contrast, LRE analysis generated E_max _estimates that correlated closely to that derived from a standard curve, despite the fact that standard curve analysis is founded upon exponential mathematics. This paradoxical result implies that the quantitative efficacy of positional-based analysis relies not upon the exponential character of real-time PCR, but instead on the ability to precisely define the relative position of an amplification profile.

**Conclusion:**

In addition to presenting insights into the sigmoidal character of the polymerase chain reaction, LRE analysis provides a viable alternative to standard curves for amplification efficiency determination, based on analysis of high-quality fluorescence readings within the central region of SYBR Green I generated amplification profiles.

## Background

All commercial real-time quantitative PCR platforms currently rely on defining the relative position of amplification profiles. As such, they are reliant on amplification of a serially diluted target to provide an estimate of amplification efficiency, which is essential to accurate and reliable quantification [1,2]. However, a major caveat of this approach is that sample-specific inhibitors can compromise both the reliability and accuracy of an assay. This can be a major concern, particularly for samples derived from sources known to contain PCR inhibitors, in that any loss of amplification efficiency will generate unidentified and potentially large quantitative errors [3,4].

Driven by the highly desirable ability to assess amplification kinetics within individual PCR reactions, a large number of studies have attempted to utilize the kinetics of amplicon DNA accumulation as the basis for amplification efficiency determination, through the application of exponential mathematics [5-15]. Derived from the intuitive notion that PCR amplification is inherently exponential in nature, this approach attempts to exploit the presence of a "log-linear region" within the lower region of real-time amplification profiles. Founded on the presumption that log-linearity reflects constant amplification efficiency, amplification efficiency is calculated from the slope of the log-linear region, similar to that conducted for standard curves.

An alternative approach to fluorescence analysis is based upon the presumption that PCR amplification is inherently sigmoidal in nature, allowing amplification efficiency to be estimated by fitting fluorescence readings to the classic Boltzmann sigmoid function using nonlinear regression analysis [16-26]. Unfortunately, effective implementation of this approach has been impeded by errors produced by distortions within the plateau phase [18,23-25]. Recent development of a simplified approach to sigmoidal analysis based on recognition that amplification rate is linearly coupled to amplicon DNA quantity, circumvents such plateau phase anomalies. Called "linear regression of efficiency" or LRE analysis, amplification efficiency is determined by applying linear regression analysis to the fluorescence readings within the central region of an amplification profile [27].

Under the LRE model, amplification efficiency is maximal at the onset of thermocycling, with amplification rate progressively slowing as amplicon DNA accumulates, such that each cycle produces a unique amplification efficiency, with entry into the plateau phase occurring as amplification efficiency approaches zero. As such, amplification efficiency is defined as the maximal efficiency (E_max_) produced in the absence of amplicon DNA. Although this clearly conflicts with the exponential model of real-time qPCR, which dictates that amplification efficiency is constant, it is unclear as to what extent these opposing interpretations impact the efficacy of amplification efficiency determination.

A central objective of this study was to critically evaluate exponential- and sigmoidal-based fluorescence analysis for determining amplification efficiency, with the expectation that standard curves would provide a gold standard from which to base the comparison. Notwithstanding the extensive resources required for their construction, the positional analysis upon which standard curves are based proved to be an effective platform from which to conduct the analysis.

## Results and discussion

### C_t_-based standard curve analysis

**Figure 1:**
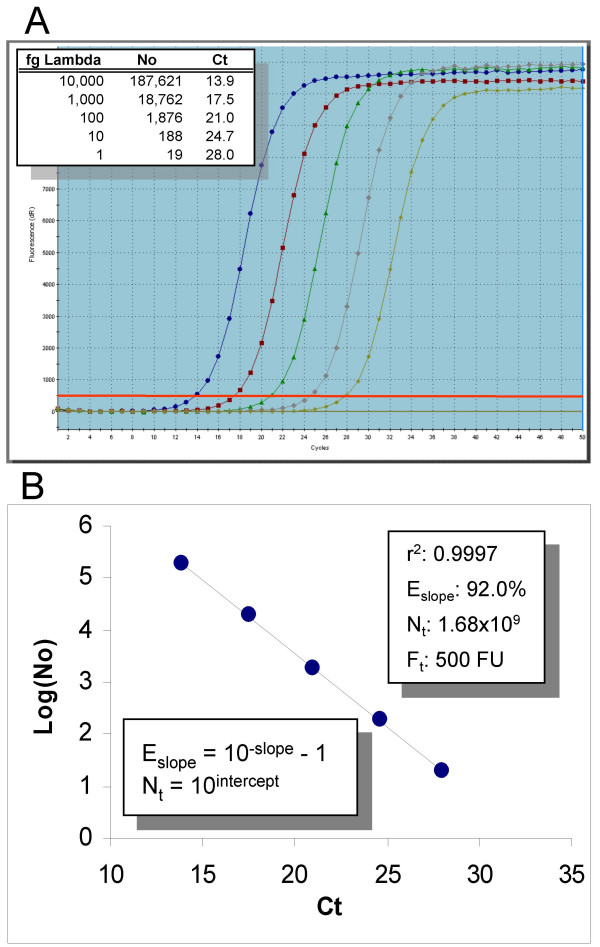
**Amplification efficiency determination via standard curve analysis**. (A) Screenshot of the amplification profiles generated by five quantities of lambda gDNA, ranging from 188,000 to 19 genomes in 10-fold increments. The fluorescence threshold is represented by the red line spanning the lower region of the profiles. Note that each amplification profile was produced by averaging the fluorescence readings of three replicate amplification reactions. (B) Plotting Log target quantity vs. C_t _generates a line in which the amplification efficiency is derived from the slope (E_slope_) and the number of amplicon molecules at threshold (N_t_) is derived from the intercept via linear regression analysis [2]. fg Lambda: femtograms of lambda gDNA, N_0_: the number of lambda genomes, C_t_: the threshold cycle, r^2^: linear regression correlation coefficient, F_t_: the fluorescence threshold, FU: fluorescence units

The current gold standard for conducting amplification efficiency determinations is based on analysis of a serially diluted target. An example of this approach is presented in Figure 1 in which lambda gDNA is diluted in 10-fold increments to cover a quantitative range of five magnitudes. Examination of the resulting amplification profiles illustrates the central principle underpinning real-time qPCR, which is that profile position is precisely related to target quantity, as reflected by the regular spacing of these amplification profiles (Figure 1A). A prominent approach to positional analysis, called the threshold method, exploits this principle by defining profile position in terms of the fraction cycle (called the threshold cycle or C_t_) at which reaction fluorescence reaches an arbitrary quantity (called the fluorescence threshold or F_t_). Target quantification can then be achieved by analyzing the position of the amplification profile generated by a sample, in relation to that produced by a serially diluted, target-specific quantitative standard.

The mathematics of the threshold method is relatively simple, originating from the premise that PCR amplification is exponential. As such, target quantity can be expressed in terms of the threshold and amplification efficiency as:

(1) $$N_{0}=\frac{N_{t}}{{\left(E+1\right)}^{C_{t}}}$$

where *N*_*0 *_is the number of target molecules, *N*_*t *_is the number of amplicon molecules at threshold and *E *is the amplification efficiency. Target quantity can thus be calculated once values for *N*_*t *_and *E *have been obtained, which can be accomplished by constructing a standard curve in which Log(N_0_) is plotted against C_t_, such that amplification efficiency is defined by the slope:

(2) *E*_*slope *_= 10 ^*slope *^- 1

where *E*_*slope *_is the slope-derived estimate of amplification efficiency [2]. Figure 1B illustrates this approach, which produced an *E*_*slope *_of 92.0% with a linear correlation coefficient (r^2^) of 0.9997.

Such a high level of linearity is consistent with the contention that PCR amplification is exponential in nature, which in turn supports the presumption that amplification efficiency is invariant. Having said this, however, it is also evident from the sigmoidal shape of a typical SYBR Green I real-time profile that amplification efficiency is not completely invariant, as reflected by the eventual cessation of amplification that defines the end point of the amplification process, commonly referred to as the "plateau phase" (Figure 1A). This potential contradiction has led many prominent studies to surmise that an "exponential region" exists within the lower domain of amplification profiles [6,9,10,13-15,20,28-30], with the inference that the upper boundary of this exponential region is defined as the point at which the amplification efficiency begins to decrease.

**Figure 2:**
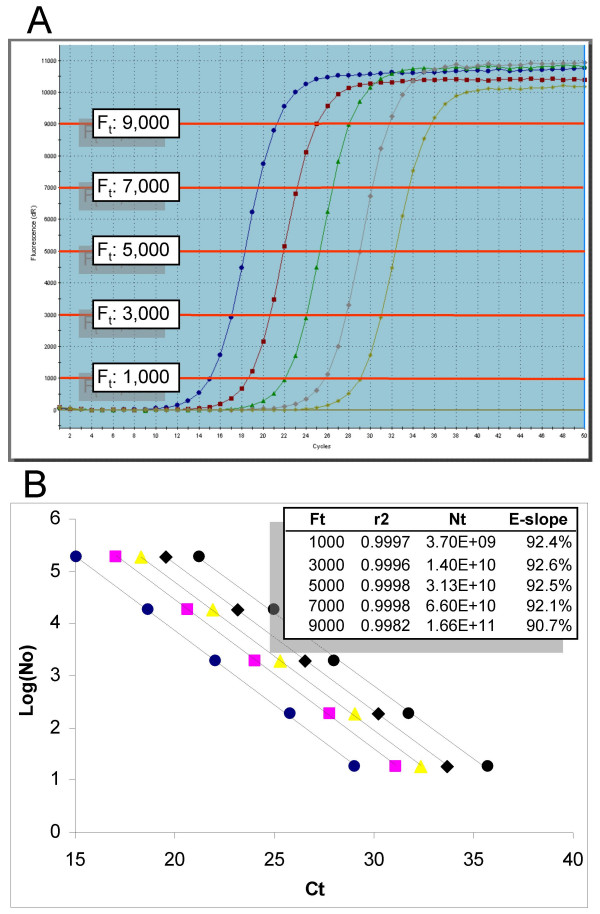
**Assessing the impact of the fluorescence threshold on slope-derived determination of amplification efficiency**. (A) Amplification profiles taken from Figure 1A with the red lines representing the fluorescence thresholds that were manually set to values that span the entire height of the amplification profiles. (B) Standard curves generated from each of the five F_t _settings (increasing from left to right). The numerical inlay summarizes the linear regression analysis, which indicates that F_t _has no major impact on either r^2 ^or E_slope _until placed into the extreme upper region of the amplification profiles.

The general validity of this presumption can be tested empirically by compiling a series of standard curves generated by progressively increasing F_t_. This also allows testing of another general presumption, which is that the integrity of a standard curve relies on placing F_t _within this putative exponential region [14,15,28,29]. As illustrated in Figure 2, this appears not to be the case, in that E_slope _was unaffected until the fluorescence threshold was placed into the extreme upper region of the profiles.

Not only does this contest the existence of an exponential region, it also challenges the perception that the efficacy of the threshold method is reliant on the exponential character of real-time amplification profiles. Partial resolution to this apparent paradox can be gained by considering PCR amplification to be an inherently sigmoidal process in which amplification efficiency is dynamic [18,27]. Under such a scenario, it could be surmised that the efficacy of the threshold method is derived from the application of a purely positional-based analysis. A slope-based estimate of amplification efficiency (E_slope_) would thus reflect the rate of amplification, not at C_t_, but rather the maximal amplification efficiency (E_max_) as defined under a sigmoidal paradigm (see below). Indeed, sigmoidal analysis provides insights that support such an interpretation.

### Kinetic-based sigmoidal analysis

**Figure 3:**
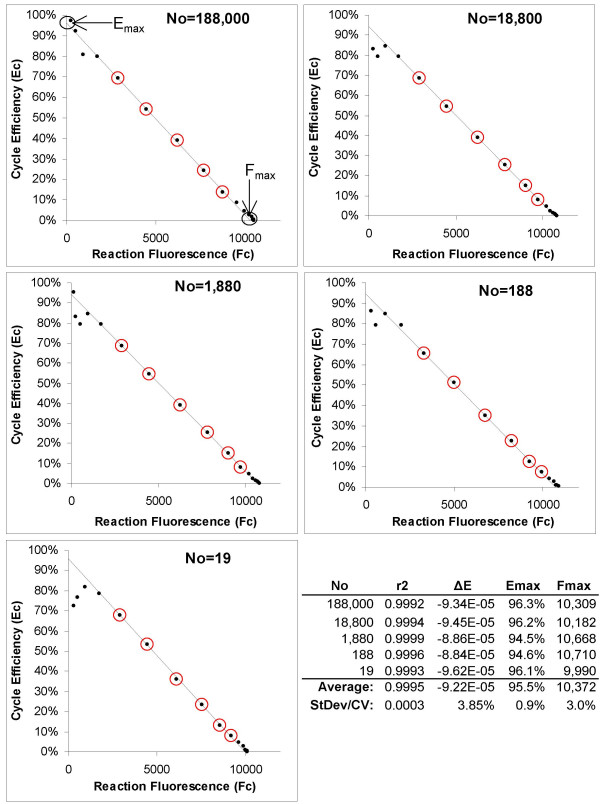
**Amplification efficiency determination via LRE analysis**. As described in detail in an earlier study [27], LRE analysis produces a linear representation of PCR amplification in which amplification efficiency (E_C_) is linearly coupled to amplicon quantity (F_C_). Best exemplified by cycles within the central region of an amplification profile, this linear relationship allows the application of linear regression analysis to a 4–6 cycle window (designated by red circles) from which amplification efficiency is determined from the Y-intercept, defined as the maximal amplification efficiency (E_max_, equation 3). As amplicon DNA reaches detectable quantities, amplification rate decreases substantively as defined by the slope (ΔE, equation 3), with the reaction fluorescence reaching a maximum (F_max_, X-intercept) as amplification rate approaches zero. The low variances produced by LRE analysis, as summarized in the tabular inlay, reflects the high level of similarity in profile shape produced by each of the five target quantities.

Recognition that amplification rate is linearly correlated with amplicon quantity led to the proposal that the dynamics of PCR amplification can be described by the linear equation:

(3) *E*_*C *_= *ΔE *× *F*_*C *_+*E*_max_

where *E*_*C *_is the amplification efficiency produced during cycle C (referred to as "cycle efficiency"), *F*_*C *_is the reaction fluorescence at cycle C and is proportional to the mass of amplicon DNA in the reaction, *ΔE *is the rate of loss in amplification efficiency and *E*_*max *_is the maximal amplification efficiency, that is, when *F*_*C *_= 0 [27]. Called "linear regression of efficiency" or LRE analysis, this approach is implemented by first estimating cycle efficiency from the relative increase in reaction fluorescence over each individual cycle:

(4) $$E_{C}=\frac{F_{C}}{F_{C-1}}-1$$

where *F*_*C-1 *_is the fluorescence reading produced by the previous cycle. Plotting E_C _against F_C _produces a line as described by equation 3, from which *E*_*max *_is determined from the intercept. Application of LRE analysis to the amplification profiles presented in Figure 1 produced an average E_max _of 95.5% ± 0.9% (Figure 3), as compared with the 92.0–92.6% E_slope _estimates produced from the standard curve analysis (Figures 1B and 2B).

**Figure 4:**
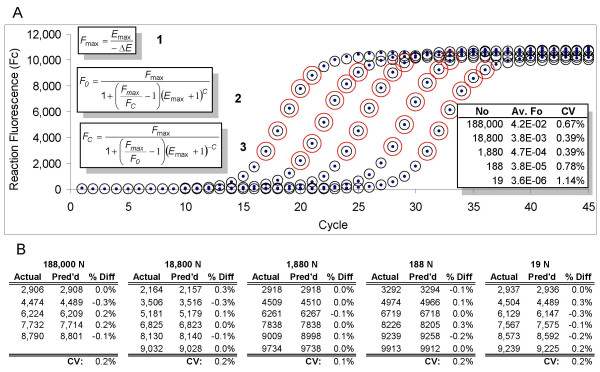
**LRE modeling of real-time PCR**. (A) The three inlaid equations allow PCR amplification to be modeled using *ΔE *and *E*_*max *_values derived from LRE analysis of cycles within the central region of each respective amplification profile (large red circles that correspond to the red circles in Figure 3). Following LRE analysis, each respective fluorescence reading (F_C_) is converted into target quantity (F_0_) using the second equation, from which an average F_0 _is calculated for each respective profile from the cycles included in the LRE analysis (Av. Fo). Reaction fluorescence can then be predicted using the third equation (black circles) that correlate closely to the actual fluorescence readings (dots). (B) Tabular summaries of the actual and predicted (Pred'd) reaction fluorescence for the cycles included in the LRE analysis, for each of the five amplification profiles. Percent difference (%Diff) illustrates the extraordinary precision that can be achieved, producing an average difference of < ± 0.2%. CV: coefficient of variation = standard deviation/average ×100%.

In addition to greatly simplifying amplification efficiency determination, development of LRE analysis was also instrumental to the derivation of two equations that allow real-time PCR amplification to be mathematically modeled. As described previously [27], the LRE model was developed by using *ΔE *and *E*_*max *_to adapt the classic Boltzmann sigmoid function to PCR. Although the primary application of LRE modeling is target quantification, it also allows PCR amplification to be predicted with a very high degree of precision, as is illustrated in Figure 4.

**Figure 5:**
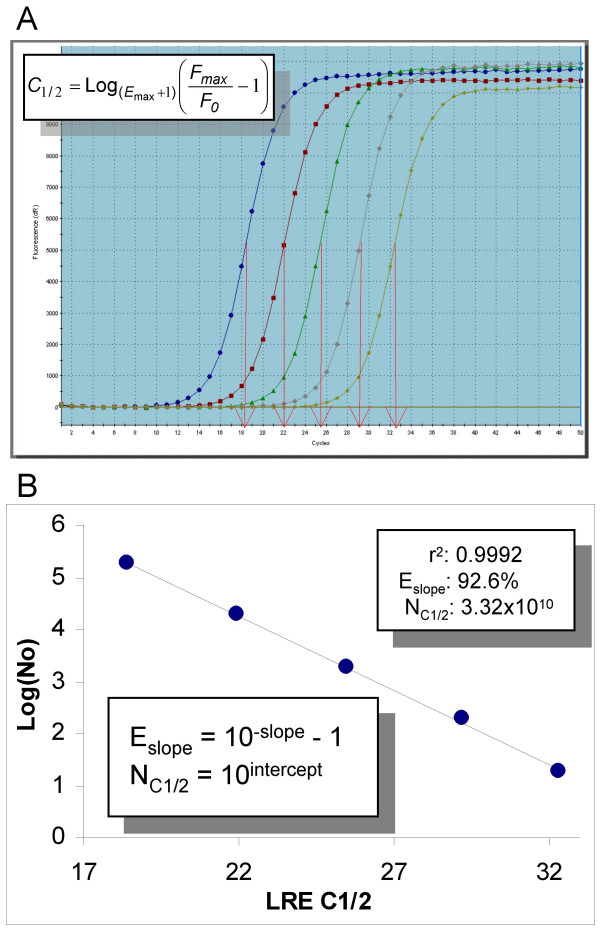
**Positional analysis via C_1/2_**. (A) Profile position is defined by the fractional cycle (designated by the red arrows) at which reaction fluorescence reaches half of maximal (F_max_). Calculated using the inlaid equation, C_1/2 _is derived from sigmoidal analysis of each individual amplification profile, rather than from defining a common fluorescence threshold as is done for determining C_t_. C_1/2 _thus provides positional information that is unique to each individual amplification profile. (B) Identical to the standard curve analyses presented in Figures 1B and 2B, amplification efficiency is derived from the slope and the number of amplicon molecules at C_1/2 _(N_C1/2_) derived from the intercept.

The LRE model can also be used to further evaluate the positional analysis upon which the threshold method is based, through the ability to describe profile position via the fraction cycle, called C_1/2_, that defines the mid-point at which reaction fluorescence reaches precisely half of its maxima [27]. C_1/2 _can be calculated for an individual amplification profile once values for *ΔE*, *E*_*max *_and average *F*_*0 *_have been obtained, using a derivative generated during development of the LRE method (Figure 5A).

Although C_1/2 _may appear to be similar to C_t_, a central attribute of C_1/2 _is that it is purely sigmoidal in origin. This then provides the opportunity to examine the efficacy of positional analysis by constructing a standard curve using the C_1/2 _values from each of the amplification profiles presented in Figure 1A. As presented in Figure 5B, this produces a slope-derived amplification efficiency estimate of 92.6%, a value that falls within the range of estimates of 92.0–92.6% produced by the C_t_-based standard curve analyses (Figures 1B and 2B). This further supports the contention that the efficacy of the threshold method is dependent on accurately defining profile position, rather than on the exponential nature of real-time PCR.

### Exponential analysis of the log-linear region

**Figure 6:**
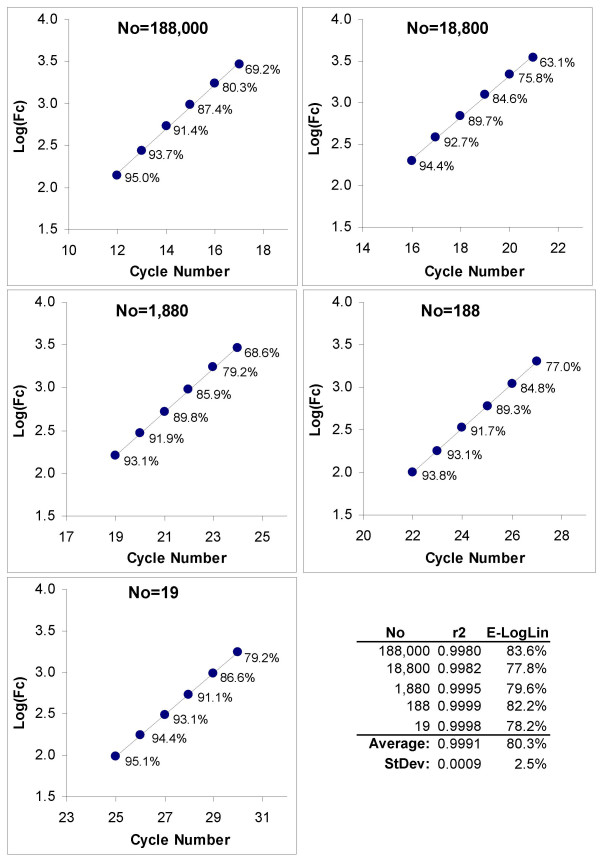
**Amplification efficiency determination based on the log-linear region using a six cycle window**. Log of the fluorescence readings within the lower region of the five amplification profiles presented in Figure 1A are plotted again cycle number, revealing a large log-linear region within all five profiles. The slope of this line provides an exponential-based estimate of amplification efficiency (E_loglin_, equation 5) as summarized in the numerical inlay. The percentages listed below each data point show the predicted cycle efficiency derived from LRE analysis (equation 3). This predicts that a large loss in amplification efficiency occurs within the log-linear region.

First introduced during the early history of quantitative PCR, a number of studies have attempted to determine amplification efficiency by exploiting the existence of a "log-linear region" within the lower region of an amplification profile [5-9]. Differing primarily in the number and position of the cycles included in the analysis, several implementations of this approach have been reported for real-time qPCR [9-15], which have subsequently been utilized for large-scale gene expression profiling in which standard curve construction is impractical [20,31,32]. Similar to the exponential mathematics of the threshold method, amplification efficiency is calculated from the slope of the log-linear region:

(5) *E*_*loglin *_= 10^slope ^- 1

where *E*_*loglin *_is the log-linear estimate of amplification efficiency. It is important to note, however, that the validity of this approach rests on the supposition that amplification efficiency remains constant throughout the log-linear region. Indeed, the very existence of a log-linear region lends itself to the compelling, albeit implicit, contention that amplification efficiency is invariant within this region. Nevertheless, critical examination reveals that linearity alone is insufficient to validate this contention. Figure 6 provides an example of this approach, based upon analysis of the five amplification profiles presented in Figure 1.

This generated an average r^2 ^of 0.9991, corroborating the presence of a substantive log-linear region within the lower region of all five amplification profiles. Nevertheless, the first indication of an anomaly is provided by the E_loglin _values produced by the five profiles, which averaged 80.3% ± 2.5% (Figure 6). This is substantially lower than the C_t_-E_slope _of 92.0–92.6%, E_max _of 95.5%, and C_1/2_-E_slope _of 92.6% generated from the same five amplification profiles (Figures 1B and 2B, 3, and 5B, respectively).

An essential insight into the nature of the log-linear region is provided by LRE analysis, in that cycle efficiency can be calculated from the reaction fluorescence using equation 3. This predicts that a 15–30% loss in amplification efficiency occurs across the six cycles included in this analysis, despite the high level of linearity (Figure 6). In addition to challenging the contention that the log-linear region is representative of an exponential region, this predicted reduction in amplification efficiency would be expected to reduce the slope as compared with an invariant amplification efficiency, which in turn would reduce the resulting E_loglin _values. Based on comparison with the "gold standard" E_slope _values, this indeed appears to be the case. Ultimately, however, conflicting interpretations raise the question as to what is the true origin of the log-linear region.

### Resolving the origin of the log-linear region

**Figure 7:**
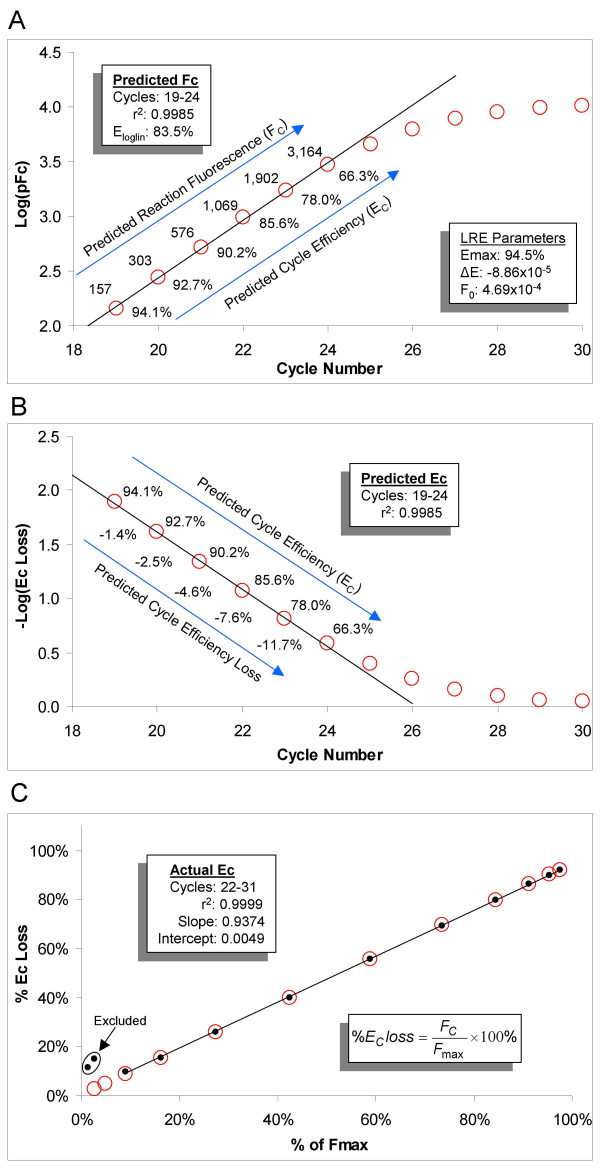
**The dynamics of amplification efficiency under a sigmoidal paradigm**. (A) A Log(F_C_) vs. cycle number plot of the predicted sigmoidal F_C _dataset taken from the 1,880 molecule profile presented in Figure 4. This reveals a log-linear region that is nearly identical to that produced by the actual F_C _dataset (Figure 6). The numerical inlay summarizes the results of the linear regression analysis. Numbers above and below the plot are the respective F_C _and E_C _values predicted for each cycle. (B) Plotting -Log of the loss in cycle efficiency vs. cycle number produces a linear region, illustrating an exponential loss in amplification efficiency within the log-linear region. (C) Plotting the loss in amplification efficiency against reaction fluorescence expressed as a percentage of F_max _generates a line, reflective of the linear relationship between amplification efficiency and amplicon quantity. This provides a simple method for estimating the loss in amplification efficiency based on expressing reaction fluorescence as a percentage of F_max_. Close correlation between the predicted (red circles) and actual (dots) values is maintained down to the point at which read precision is compromised by low reaction fluorescence, as reflected by the two lowest actual E_C _values. The numerical inlay summarizes the results of the linear regression analysis based on the actual E_C_, excluding the two lowest values.

The sigmoidal modeling presented in Figure 4 provides the opportunity to examine the characteristics of the log-linear region under a purely sigmoidal paradigm. Importantly, this also allows the dynamics of PCR amplification to be examined within the lowest regions of an amplification profile, where low reaction fluorescence intensity precludes effective E_C _determination using equation 4. Figure 7A presents a classic Log(F_C_) vs. cycle number plot of a sigmoidal F_C _dataset taken from Figure 4.

This confirms the presence of a log-linear region that generates an E_loglin _estimate of 83.5%, similar to the 79.6% E_loglin _estimate generated from analysis of the actual F_C _readings (Figure 6). LRE modeling predicts a large loss in amplification efficiency within this log-linear region in close agreement with that predicted from the actual F_C _dataset (Figure 6). Another key attribute of this sigmoid-derived log-linear region is illustrated in Figure 7B, which is that the loss in amplification efficiency is exponential in nature, suggesting that it is an exponential loss in amplification efficiency that underpins the linear character of the log-linear region.

Additional perspectives on the kinetics of real-time PCR are provided by Figure 7C, which demonstrates that loss in amplification efficiency can be estimated by expressing reaction fluorescence as a percentage of F_max_. For example, a reaction fluorescence that is 1% of F_max _corresponds to an approximate 1% loss in amplification efficiency. Importantly, this provides a simple method for illustrating at what point loss in amplification efficiency becomes nontrivial. In relation to SYBR Green I detection under the optical capabilities of the instrumentation used in this study, 1% of F_max _corresponds to about 100 fluorescence units, which is below the lowest fluorescence intensity that can be measured with acceptable accuracy. Accordingly, at 1,000 fluorescence units, which roughly corresponds to the middle of the log-linear region, the loss in amplification efficiency is estimated to be 10%. It would thus be difficult to obtain accurate fluorescence readings in which loss in amplification efficiency can be considered to be trivial, without a substantive increase in detection sensitivity. Of greater significance, however, is that a measurable loss in amplification efficiency occurs within 99% of a real-time amplification profile, irrespective of detection sensitivity, refuting the exponential character that has historically been ascribed to real-time PCR.

### Comparison of automated data processing packages

Although detailed analysis of a single standard curve may be sufficient to demonstrate lack of exponential character, it only provides a limited perspective as to the potential impact on currently employed data processing packages. The evaluation was therefore expanded to include four identical standard curves in which the reaction mixes were supplemented with increasing quantities of SYBR Green I. Initially based on concerns that SYBR Green I quantity could impact the quantitative accuracy of LRE modeling [27], this approach also provides some perspective as to how each method responds to the inhibition of amplification, produced by increasing SYBR Green I quantity. It should also be noted that DyNAmo (formulated with an engineered *T. brockianus *DNA polymerase fused to a non-specific DNA-binding region) was used for this analysis, demonstrating that the trends described here are not unique to enzyme formulations containing *T. aquaticus *DNA polymerase.

The evaluation was also extended to include three publicly available software packages that provide automated amplification efficiency determination, two of which are based on analysis of the log-linear region. A major challenge for both of these exponential-based packages is selecting which cycles to include in the analysis. The first, called "LinReg", uses an iterative method that searches for the greatest linear correlation coefficient based on a minimum four cycle window, in which amplification efficiency is determined from the slope [11], as is used in this study for E_loglin _determination. The second, called "Miner", uses a complex, multi-step approach in which cycle selection is based on a series of calculations using nonlinear regression to a four-parameter logistic model to define the upper boundary of the analysis. This is followed by fitting the selected fluorescence readings to an exponential equation using an iterative nonlinear regression analysis approach, from which amplification efficiency is determined using a weighted average [15]. The third is a prototypic Java program that automates LRE analysis, which is provided as supplementary material by Rutledge and Stewart 2008 [27]. The results of the evaluation are summarized in Table 1.

**Table 1:** Comparison of amplification efficiency determinations generated by six different methodologies, based on four replicate standard curves

	**Positional Analysis (Standard curve)**		**Fluorescence Analysis (Individual amplification profiles)**			
	***Serial Dilution (n = 6)***		***Sigmoidal***	***Exponential***		
	***Linear Regression (E*_*slope*_*)***		***Linear Regrn***	***Linear Regression***		***Nonlinear Regrn***
***[SG]***	***C*_*t*_-*E*_*slope*_**	***LRE C*_*1/2*_-*E*_*slope*_**	***LRE E*_*max*_** ***(n = 6)***	***E*_*loglin*_** ***(n = 6)***	***"LinReg" (n = 6)***	***"Miner" (n = 6)***
0.5X	98.8% (0.9993)	97.9% (0.9999)	96.0% ± 1.7%	81.1% ± 1.6%	87.5 ± 3.5%	93.0 ± 1.8%
1.0X	96.2% (0.9994)	95.7% (0.9990)	95.6% ± 1.3%	79.8% ± 1.6%	89.6 ± 3.0%	90.6 ± 2.1%
1.5X	94.1% (0.9990)	93.5% (0.9995)	92.8% ± 0.9%	83.1% ± 1.4%	84.6 ± 4.2%	90.5 ± 2.8%
2.0X	90.9% (0.9989)	89.7% (0.9991)	85.6% ± 0.7%	79.2% ± 2.1%	90.8 ± 3.8%	87.2 ± 1.7%

Amplification efficiency was determined using four replicate standard curves supplemented with increasing quantities of SYBR Green I. The data is categorized based on the type of analysis. The bracket numbers under positional analysis are the linear correlation coefficients (r^2^) generated from each standard curve. The fluorescence analysis lists the respective average amplification efficiency ± standard deviation for the six target quantities used in the standard curve construction. [SG]:quantity of supplementary SYBR Green I, n: the number of profiles used in the construction of the standard curves.

Similar to that observed previously, positional analysis produced a high level of correlation between C_t_- and C_1/2_-based E_slope _values. Both present a progressive loss in E_max _as SYBR Green I quantity was increased, resulting in an 8% reduction at the highest quantity examined. Increasing SYBR Green I quantity had no apparent impact on the r^2 ^of each standard curve, indicating that increasing reaction fluorescence did not improve the precision of the analysis.

As predicted by sigmoidal modeling (Figure 7) all three methods that use exponential analysis produced lower efficiency values, although the magnitude of this difference varied. Potentially more significant is that exponential analysis generated no discernible trend when SYBR Green I quantity was progressively increased, with the possible exception of Miner, which predicted a 6% loss at 2.0X SYBR Green I. It is important to also note that exponential analysis is highly influenced by the number and position of the cycles included in the analysis. This is particularly evident for LinReg in which cycle selection is based on searching for a maximal r^2 ^using a variable cycle window size and position. This can produce large variances and frequently requires manual adjustment. However, regardless of implementation, the invalidity of applying exponential mathematics to the log-linear region brings into question both the reliability and accuracy generated by LinReg and Miner.

Consistent with that seen in Figure 3, automated LRE analysis produced E_max _values similar to the E_slope _values, except at the highest SYBR Green I quantity, which produced a ~4% lower efficiency estimate. Notwithstanding this discrepancy, this dataset does provide substantive supporting evidence that LRE analysis generates amplification efficiency estimates that correlate more closely to E_slope_, and generate less variance than methods that rely on exponential analysis of the log-linear region.

## Conclusion

Kinetic analysis of PCR amplification based on the LRE model reveals fundamental flaws in the current interpretation of amplification efficiency dynamics, demonstrating that SYBR Green I-derived amplification profiles lack the exponential character that has historically been ascribed to real-time PCR. Consequently, methods for amplification efficiency determination that rely on exponential analysis of the log-linear region generate systematic underestimations, differing only in the extent of the bias and variability in the resulting amplification efficiency estimates. Although this apparent lack of exponential character could also be expected to compromise the efficacy of the threshold method, empirical testing demonstrates that positional analysis does not rely on the exponential character of a real-time amplification profile. Furthermore, it was demonstrated that the slope-derived efficiency estimate produced by C_t_-based standard curves reflect the amplification efficiency not at C_t_, but rather the maximal amplification efficiency as defined under a sigmoidal paradigm.

This study further corroborates the efficacy of LRE analysis for amplification efficiency determination, as well as providing additional insights into the linear coupling between amplification efficiency and amplicon DNA quantity. LRE analysis thus not only provides a gateway to sigmoid-based quantification, but also provides a simple methodology for analyzing amplification kinetics within individual amplification reactions. Based on analysis of the high-quality fluorescence readings within the central region of an amplification profile, LRE analysis avoids errors associated with both low reaction fluorescence and distortions associated with the plateau phase.

## Methods

PCR amplification was conducted as previously described [27] in which 3–4 replicate reactions were run for each quantity of lambda gDNA, and the F_C _datasets averaged to generate a single amplification profile for analysis. Briefly, replicate amplification sets consisting of 5.0 μl reactions containing lambda gDNA (New England BioLabs) at the specified quantity and 500 μM of the lambda primers K7B (CTGCTGGCCGGAACTAATGAATTTATTGGT) and K12 (ATGCCACGATGCCTCATCACTGTTG). The standard curve presented in Figure 1 employed QuantiTect (Qiagen) enzyme formulation, whereas DyNAmo (Finnzymes, distributed by New England BioLabs) was used for the standard curves containing increasing quantities of SYBR Green I (Table 1). SYBR Green I was diluted to the appropriate quantity using ddH_2_0 before addition to the PCR master mix just prior to amplification reaction preparation and is expressed in units designated by the manufacturer (Invitrogen).

All amplifications were conducted with a Mx3000P spectrofluorometric thermal cycler (Stratagene) using a two temperature cycling regime initiated with a 15 min activation at 95°C, followed by 50 cycles of 120 s annealing and elongation at 65°C and a 10 s denaturation at 95°C. To increase optical precision, three fluorescent reads were taken at the end of the annealing and elongation step and the average used as an estimate of reaction fluorescence. Specificity of amplification was confirmed by melting curve analysis conducted at the end of each run.

An extensive description of the development and implementation of the LRE method is provided by Rutledge and Stewart [27]. Automated LRE analysis was conducted using the prototypic Java program provided as supplementary materials in this earlier study using default values.

## Authors' contributions

RGR conceived and led the project, in addition to drafting the manuscript. DS conducted all of the real-time qPCR and contributed to the data analysis.
